# The Role of Fat Reducing Agents on Adipocyte Death and Adipose Tissue Inflammation

**DOI:** 10.3389/fendo.2022.841889

**Published:** 2022-03-24

**Authors:** Ahava Muskat, Megan Pirtle, Yana Kost, Beth N. McLellan, Kosaku Shinoda

**Affiliations:** ^1^ Department of Medicine, Division of Dermatology, Albert Einstein College of Medicine, Bronx, NY, United States; ^2^ Department of Molecular Pharmacology, Albert Einstein College of Medicine, Bronx, NY, United States; ^3^ Department of Medicine, Division of Endocrinology & Diabetes, Albert Einstein College of Medicine, Bronx, NY, United States; ^4^ Albert Einstein College of Medicine, Fleischer Institute for Diabetes and Metabolism, Bronx, NY, United States

**Keywords:** phosphatidylcholine, deoxycholic acid, adipocyte, TNF - α, lipolysis, apoptosis, necrosis

## Abstract

Deoxycholic Acid (DCA), which is an FDA-approved compound for the reduction of submental fat, has evolved through an unanticipated and surprising sequence of events. Initially, it was used as a solvent for Phosphatidylcholine (PDC), which was thought to promote lipolysis, but it was later proven to be the bioactive component of the formula and is currently widely used as Kybella. It has also been used off-label to treat other types of fat deposits like lipomas, HIV lipodystrophy, and excess orbital fat. Despite widespread clinical use, there has been no consensus clarifying the mechanisms of DCA and PDC alone or in combination. Furthermore, despite PDC’s removal from the FDA-approved formula, some studies do suggest it plays an important role in fat reduction. To provide some clarity, we conducted a PubMed search and reviewed 41 articles using a comprehensive list of terms in three main categories, using the AND operator: 1) Phosphatidylcholines 2) Deoxycholic Acid, and 3) Lipoma. We isolated articles that studied PDC, DCA, and a PDC/DCA compound using cell biology, molecular and genetic techniques. We divided relevant articles into those that studied these components using histologic techniques and those that utilized specific cell death and lipolysis measurement techniques. Most morphologic studies indicated that PDC/DCA, DCA, and PDC, all induce some type of cell death with accompanying inflammation and fibrosis. Most morphologic studies also suggest that PDC/DCA and DCA alone are non-selective for adipocytes. Biochemical studies describing PDC and DCA alone indicate that DCA acts as a detergent and rapidly induces necrosis while PDC induces TNF-α release, apoptosis, and subsequent enzymatic lipolysis after at least 24 hours. Additional papers have suggested a synergistic effect between the two compounds. Our review integrates the findings of this growing body of literature into a proposed mechanism of fat reduction and provides direction for further studies.

## Introduction

Over the past 30 years, injectable fat reducing compounds have been utilized primarily for cosmetic purposes. Phosphatidylcholine (PDC) is a type of phospholipid that is a main component of cell membrane walls and a vital source for many signaling mediators (1). Intravenous PDC was originally discovered as a treatment for fat embolisms and evolved into an injectable compound with Deoxycholic Acid (DCA) as its solvent to reduce excess fat. The compound was primarily used in Europe, South America, and South Africa (2). PDC has also been studied extensively for non-cosmetic purposes and there is much-published literature describing its effect. Multiple studies show its benefit in treating hypertriglyceridemia, coronary atherosclerosis, hypothyroidism, and insulin resistance (3, 4). Additionally, PDC has been shown to be protective against hepatic fibrosis through the regeneration of hepatocytes. In patients treated with PDC, studies show noticeably elevated levels of high-density lipoproteins (HDL) and suppression of atherosclerotic plaques. Interestingly, PDC has been shown to induce inflammation through biosynthesis of prostaglandins, leukotrienes, and thromboxanes in one study, while another study showed that PDC can be protective against inflammation *via* suppression of toll-like receptor 4 (TLR-4) (5, 6). It is also known that perturbation of PDC homeostasis causes cell death by a variety of proven mechanisms while exogenous PDC can be protective against cell death in certain cases (1).

In 2002, ANVISA, the Brazilian equivalent of the FDA, banned the injection of PDC/DCA compounds for cosmetic fat reduction citing a lack of supporting evidence to prove its safety and efficacy. Subsequently, The Medicines and Healthcare products Regulatory Agency in the United Kingdom and the FDA strongly discouraged their use. Since then, many studies have been published which conclude that DCA, not PDC, is the bioactive component responsible for fat reduction. In 2015, the FDA approved DCA, as ATX-10 or Kybella (Kythera Biopharmaceuticals, subsidiary of Allergan, Westlake Village, California), for the improvement of submental fat (SMF) (7). Having excess SMF has been shown to substantially detriment patients’ self-esteem and promote specific behaviors like avoiding video conferences and wearing concealing clothing, regardless of SMF severity grade (8). A recent paper by Humphrey et al. demonstrated that DCA treatment was found to ameliorate the psychological impacts of SMF even three years after treatment (9). Both by blinded evaluators and patient surveys, treatment with DCA was considered satisfactory (9–11). DCA has also been used off-label for lipoma treatment, orbital fat reduction, and HIV/HAART-associated buccal fat pad lipodystrophy (12). Endogenous DCA is a secondary bile acid that emulsifies dietary fat in the gastrointestinal tract to allow for proper absorption (13). In recent years, DCA has been shown to cause intestinal inflammation through induction of gut dysbiosis, promote cancer cell migration in colon cancer, and function as a tumor suppressor in gallbladder carcinoma (14–16). In general, bile acids have been shown to be cytotoxic through disruption of cell and mitochondrial membranes. Specifically, DCA’s carcinogenic capability is attributed to its ability to damage chromosomes and produce reactive oxidative species in the gut (17).

While the mechanisms of action of PDC and DCA have been discussed in the literature, their mechanisms in adipocytes are not completely understood. In fact, the literature contains many conflicting papers in which some continue to suggest that PDC may actually play an important role in the reduction of adipose tissue. Additionally, there is no clear consensus regarding the specific type of cell death or lipolytic action that DCA and/or PDC induces. Moreover, although DCA has been isolated in its therapeutic capacity, it is quite important to understand the role that PDC and DCA play together and if a synergistic effect takes place when the two are compounded.

Our review is a comprehensive and broad look at the literature that studies PDC and DCA separately and together in the context of adipose tissue. We hope that this paper will elucidate current thinking and provide researchers with a future direction for further study.

## Methods

A PubMed search was conducted in October 2021 using a comprehensive list of terms in three main categories, using the AND operator: 1) Phosphatidylcholines 2) Deoxycholic Acid, and 3) Lipoma. We included articles describing the mechanistic effect of phosphatidylcholines, deoxycholic acid, or a combination of the two on fat reduction. Our search period spanned 44 years from 1977 to 2021. Because the relevant literature is limited, all available literature was reviewed.

Studies were excluded if they were not in English and if they did not include analysis of the mechanism of action of each compound using biochemical assays or histological analysis. A total of 2,713 articles were found, we excluded 2,536 because the abstracts showed that the studies were not eligible for inclusion due to a subject of study that was out of scope (studied non-adipose tissue) or was clinical in nature and did not include any biochemical assays or histology. Of the remaining 177 articles, the full texts were obtained and reviewed for eligibility criteria. Finally, 41 articles were included in this review.

## Morphological Changes

Over the past 15 years, multiple studies have explored the impact of DCA, PDC, and a combination of the two, PDC/DCA, on different types of adipose tissue using histopathology. It is important to recognize that in these publications, pathologists often used words like “inflammation”, “fat necrosis”, and “fibrosis” as descriptors of histology, but these findings may differ from the actual biochemical processes that precisely define these terms. However, it is valuable to understand how analyses of tissue through the lens of cell biology have informed our current thinking. Below, we comprehensively describe morphologic findings after injection of PDC/DCA, DCA, and PDC in chronological order to highlight both the consistent and conflicting conclusions in the literature thus far. For more details about each study, including selected histological findings, see **Table 1**.

**Table 1:** Histological Findings of PDC/DCA, DCA, and PDC injected adipose tissue.

Authors	Year	Vehicle	Subject of Study	Biopsy time post-injection	Selected histological findings
Rose and Morgan (18)	2005	Human	PDC/DCA injection into localized fat deposition on flank	1 and 2 weeks	1 week: “dense *inflammation* with plasma cells, lymphocytes, neutrophils and macrophages”.
					2 weeks: giant cells and “*fat necrosis* and adipocyte microcyst formation”.
Duncan and Hasengschwandtner (19)	2005	Human	PDC/DCA injection into abdominal fat	1 month	Gross: White nodules of *“fat necrosis”* with strands of scarred tissue.
					Microscopic: cell wall disruption, focal *inflammation* and collagen deposition. Reduction in the diameter of fat cells.
Rittes et al. (20)	2006	Rabbit	PDC/DCA into rabbit fat	4 days (4 injections per day for 4 days)	Gross: Microhard nodules
					Histologic: neutrophils, giant cells, and histiocytes
Salles et al. (21)	2006	Rabbit	PDC/DCA into rabbit fat	3, 7, 14, 21 days (weekly injections for 5 weeks)	No necrosis. Progressive *inflammatory i*nfiltrate from days 3-14, no inflammation on day 21. *Fibrosis* seen.
Kopera et al. (22)	2006	Human	PDC/DCA into human lipoma	12 weeks (3 injections over 3 weeks)	Foamy histiocytes and lymphocytes were present while no neutrophils were observed. Degeneration of fat tissue with “arabesque” structures, which represent infoldings of adipocyte membranes. Focal *fibrosis.*
Bechara et al. (23)	2007	Human	PDC/DCA into multiple lipomas	4, 10, 24, 48 hours and 10, 30 and 60 days after injection.	Early findings: destroyed and deformed adipocytes.
					Later findings: granulomatous *inflammation* with foamy histiocytes
					“encapsulated *fat necrosis”.* Thickened septa and broadened lipoma capsule. Dilated blood vessels.
Schuller-Petrovic et al. (24)	2008	Rat	PDC/DCA into rat adipose tissue	30 days (injection on day 0, 7, 28)	Lowest dose (50uL): edema, histiocytes with foamy cytoplasm
					Middle dose (300uL): bandlike fibrosis, decreased numbers of muscle fibers.
					Highest dose (600uL): “huge zones of fat *necrosis”*, hemorrhage, infiltrate involving deep subcutaneous fat, “microcysts”. Necrotic small blood vessels.
Noh and Heo (25)	2012	Rat	PDC/DCA into rat inguinal tissue	4 days	“A histiocytic and giant cell reaction are seen with fat necrosis”
Nabavi et al. (26)	2009	Human	PDC/DCA into orbital fat tissue	12 days	“Fat *necrosis”*, “lymphocytic infiltration” and “broad bands of *fibrosis.*”
Bechara et al. (27)	2012	Human	PDC/DCA into multiple lipomas	4 h, 10 h, 24 h, 48 h and 10 weeks	Adipocyte shrinkage and destruction from hour 10 up to 48-hour post-injection. Typical features of necrotic (versus apoptotic) cell nuclei were present.
Reeds et al. (28)	2013	Human	PDC/DCA injected into adipose tissue.	1 week and 8 weeks (between 2-4 injections over 8 weeks)	1 week: Macrophages and “crown like” figures (disappeared by 8 weeks).
					No changes in adipocyte diameter, volume, or lipid content.
Park et al. (29)	2013	Human	PDC/DCA into abdominal fat	6 months	Substitution of fat by *fibrosis*”, “micro abscesses”, *“inflammatory* infiltrate”, and “fat *necrosis* with microcalcification and cyst formation”, fibroid *necrosis* with extravasation in the small blood vessels.
Rotunda and Kolodney (31)	2006	Human	DCA into lipoma	2 days	Well demarcated necrosis” and “acute inflammation.”
Odo et al. (32)	2007	Human	DCA into adipose tissue	3 and 6 months	3 months: acute *inflammation*, “phagocytosis of fat cells by macrophages” and “*fat necrosis”*
					6 months: Inflammation decreased. Fibroblasts and f*ibrosis* increased.
Schuller-Petrov et al. (24)	2008	Rat	DCA and PDC/DCA into rat adipose tissue	30 days	DCA: Significant reduction in membrane integrity and cell viability to the same extent as PDC/DCA.
					Thrombus formation of vessels, necrotic vessels, muscle fiber necrosis, hair follicle necrosis and gland necrosis.
Gupta et al. (33)	2009	Rabbit	DCA and PDC/DCA into dorsal fat pad of rabbits	24 hours	DCA: Intense lysis and distortion of cell membranes
					PDC/DCA: Markedly decreased blurring of adipose cell membranes compared to DCA alone.
Duncan et al. (34)	2009	Human	DCA and PDC/DCA into abdominal fat	Variable amongst samples. (1 hour, 1 day, 1 week, 2 week, 3 weeks, 4 weeks)	1 hour/1 day: DCA samples showed immediate and massive “*fat necrosis*” compared to PDC/DCA samples.
					1 week/2 week: *fat necrosis* begins to appear in the PDC/DCA samples and a substantial, but fractionated *necrosis* is seen at two weeks.
					2 week-4 weeks: DCA samples show “moth-eaten” and “extensive eradication” of fat cells.
Thuangtong et al. (35)	2010	Mice	DCA into mice gluteal fat pads and tails	20 days (3 injections over 5 days)	Fat pads: Immune response with appearance of foamy histocytes and adipocyte destruction. Evidence of triglyceride release was noted.
					Tail findings: No impact on muscle fascicles and epidermis.
Hubner et al. (36)	2014	Human	DCA and PDC/DCA in abdominal fat	1, 3, 5, and 7 hours	Similar results of “disruption of adipocyte cell architecture” with both types of injections was seen as early as one hour post injection with maximal disruption at 5 hours. Autolysis was present at 7 hours post injection.
Safari et al. (37)	2020	Mice	DCA into mice inguinal fat pads	14 days	“crown-like structures” indicating in*flammation* and clearance of dead adipocyte cells. Increased immune infiltrate compared to control.
Walker et al. (38)	2020	Human	DCA into abdominal fat	Variable amongst samples: 1, 3, 7, or 28 days	1 day: adipocytolysis, *inflammation* and blood vessel injury
					3 days: reduced inflammation compared to day 1 with “lipid lake” formation (triacylglycerols and fatty acids surrounded by macrophages).
					7 days: extensive adipocytolysis, mild inflammation, lipid-laden macrophages
					28 days: resolved inflammation, neovascularization, atrophic fat lobules.
Palumbo et al. (39)	2010	Human	PDC versus DCA versus PDC/DCA into human adipose tissue	24 hours	DCA alone: cell membrane disruption.
					PDC and PDC/DCA: no difference from control.
Chung et al. (40)	2014	Rat	PDC versus DCA versus PDC/DCA into hind paw tissue	4 hours	PDC: mild swelling, minimal inflammatory reaction
					DCA: significant swelling and neutrophils.
					PDC/DCA: swelling to a lesser extent than DCA with migration of neutrophils.
Kim and Chung (41)	2021	Mice	PDC into inguinal fat pad	24 hours (3 injections over 3 days)	Marked adipocyte destruction and a reduction of mean size of adipocytes by 10.7% compared to controls.

### Morphologic Findings of PDC/DCA Injected Tissue

To our knowledge, Rose and Morgan, in 2005, were the first to publish morphologic findings based on skin biopsies treated with a PDC/DCA compound. They concluded that PDC/DCA works by causing adipocyte cell membrane lysis either by direct detergent effect or indirect inflammation which leads to “fat necrosis” and concludes with resorption of fat cells, fibrosis, and new collagen formation (18). Duncan et al. performed a similar study. Their findings suggested that the mechanism of action of this compound is a combination of cell-wall disruption and enzymatic transport of fatty acids and triglycerides resulting in a decreased adipocyte size (19).

In 2006, two papers were published in which a formulation of PDC/DCA was injected into rabbit fat. Rittes et al. observed “extensive fat necrosis and acute suppurative inflammation” and concluded that the formula caused immediate necrosis followed by inflammation (20). Contrasting Rittes et al.’s reports, Salles et al. determined that “necrosis” was not observed in any sample but an inflammatory infiltrate was seen progressively, increasing from days 3 to 14. The lack of necrosis observed might be due to subjective differences in how this study’s pathologists qualified histologic necrosis, but details explicating their process were not provided (21).

In 2006, two separate groups utilized PDC/DCA for lipoma reduction. Kopera et al.’s case report concluded that morphologic findings of the lipoma injected with PDC/DCA were similar to findings typically seen after general injections of foreign substances into subcutaneous fat suggesting that the effects of the injection were not a reaction to the product itself (22). In line with this, Bechara et al. published a more comprehensive paper on PDC/DCA injections into multiple lipomas. Notably, at only four hours, smaller, deformed and destroyed adipocytes were noted. The author concluded that this reaction may be considered “factitial panniculitis” which is a general induction of inflammation after injection of a foreign substance (23).

In 2008, a study was performed in which injections of increasing doses of PDC/DCA were administered into rat adipose tissue. Importantly, in some regions, muscle fibers disappeared and the walls of small blood vessels were necrotic. At the highest dose, traumatic fat necrosis was seen at the level of subcutaneous tissue. Similarly, in the human tissue studied, the highest dose produced “necrosis of fat and small vessel disruption”, suggesting a non-selective action of PDC/DCA (24). Like Schuller-Petrovic et al., Noh and Heo studied PDC/DCA on 15 rat’s inguinal fat pads and concluded that there was a significant increase in “inflammatory activity”, “necrosis”, and “fibrosis” compared to controls (25).

Utilizing a different study subject, Nabavi et al. published a case report in which morphologic features of orbital fat injected with PDC/DCA were described. The authors stated that it is feasible to define PDC/DCA’s mechanism of action as inducing “fat necrosis” as findings included variably sized adipocytes engulfed by multinucleated giant cells, which is characteristic of the process of necrosis (26).

More recently, Bechara et al.’s group deduced that the mechanism of action of PDC/DCA was necrosis based on electron microscopy, which is considered the “gold standard” to differentiate apoptosis from necrosis. Additionally, most adipocytes remained negative for active caspase-3, but stained positive for TUNEL and DNase I, supporting necrosis as opposed to apoptosis as the mechanism of PDC/DCA (27).

Contrasting earlier reports, Reeds et al. found no changes in adipocyte size, volume, or lipid content when they injected PDC/DCA into adipose tissue. However, findings of macrophages and crown-like structures which are hallmarks of adipocyte death support necrosis as the mechanism of PDC/DCA (28). Park et al. studied adipose tissue results six months after injection, which was the longest time post-injection seen in the reviewed literature. Their morphologic findings were similar to earlier reports. Like Schuller-Petrovic et al., they also noted fibroid necrosis with extravasation in the small blood vessels suggesting non-selectivity of the compound (29).

Of note, not only have morphologic studies of PDC/DCA injections shown consistent pathological evidence of inflammation, but one group studied the molecular characteristics of the inflammatory pathway following PDC/DCA injections. They found that inflammatory cytokines, TNF-α, IL-6, IL-8, and IL-10 mRNA levels were significantly elevated 48 hours after treatment (30).

In conclusion, morphologic reports describing findings after PDC/DCA injections most often contain features of “necrosis”, “fibrosis”, and “inflammation”. Some studies noted a decrease in adipocyte size or shape (19, 23), but one study (28) specifically noted no difference. A few studies also noted non-adipocyte reactions to PDC/DCA (24, 29) suggesting its non-selectivity. Two early studies suggested that PDC/DCA induced a general reaction that typically occurs with foreign substances (22, 23). Most studies indicated that PDC/DCA injections induced necrosis with accompanying inflammation without any findings of “apoptosis” or “lipolysis”. These findings have been consistent throughout the years, even with variability amongst sample size, subject, number of injections, and length of study.

### Morphologic Findings of DCA Injected Tissue

In trying to clarify the roles of PDC and DCA separately, it is valuable to review morphologic findings of tissue that have been injected with purified samples of either. The first study we found in this category was in 2006 by Rotunda and Kolodney, who injected DCA into a lipoma. Like with PDC/DCA injections, “well-demarcated necrosis” and “acute inflammation” were seen (31). In 2007, Odo et al. found that pure DCA injections resulted in a substitution of fat by fibrosis (32). Schuller-Petrovic et al. compared the morphologic results of PDC/DCA versus DCA alone on rat models. They found that DCA alone significantly reduced membrane integrity and cell viability to the same extent as PDC/DCA, suggesting that DCA is the bioactive component in the formulation, as opposed to PDC. Their findings also suggested that DCA does not act selectively on adipocytes (24).

Similarly, Gupta et al. studied PDC/DCA versus DCA injections alone. Interestingly, they found that the tissue treated with PDC/DCA showed markedly decreased blurring of adipocyte membranes compared to DCA. The authors postulated that this is due to different dispersion qualities between the two formulas and will be described later in this review. Like the previous reports, they concluded that DCA is the bioactive component of the formulation (33). In 2009, a serial histological study was published comparing PDC/DCA injections to DCA injections into the abdomen and skin. The findings were significant for gradual necrosis in the PDC/DCA samples versus immediate necrosis (seen after only one hour of treatment) in the DCA sample. Again, they show that DCA is the main bioactive component (34).

In another study by Thuangtong et al., the authors studied mouse tails to assess the specificity of DCA for adipocytes, or if like Schuller-Petrovic et al. suggested, DCA induced general necrosis and was non-selective. This group found that DCA was selective as muscle fascicles and epidermis were unaffected in the tissue samples (35). This difference might be attributed to the concentration of DCA utilized in the different papers (2.5% versus 0.5% DCA).

Hübner et al. found no significant difference in adipocyte morphology comparing PDC/DCA versus DCA injected tissue. Importantly, based on positive tumor necrosis factor-alpha (TNF-α) (indicative of necrosis) and negative caspase-3 (indicative of apoptosis) staining, they deduced that the mechanism of action of DCA is “necrosis” and not “apoptosis”. The same results were seen in PDC/DCA treated samples indicating again that DCA is the bioactive component of the compound (36). Recent studies have remained consistent with previous reports, showing that DCA induces inflammatory cell death and that DCA is non-selective for adipocytes. Specifically, vascular injury was noted at higher dose concentrations (2% and 4%) which is consistent with Schuller-Petrovic et al.’s findings above (37, 38).

### Morphologic Findings of PDC Injected Tissue

In our review, we identified only three reports of purified PDC injection into adipose tissue. In 2009, Palumbo et al. compared PDC versus DCA versus PDC/DCA injections in human adipose tissue. Interestingly, they found that compared to DCA alone, PDC and PDC/DCA injected adipose tissue appeared very similar to control tissue, indicating that PDC may be protective against DCA (39).

Next, through analysis of rat tissue and utilizing immunohistochemical markers for inflammation (IL-6, IL-1β, PGE-2, and MPO), Chung et al. found that PDC treated tissues appeared no different than untreated tissues. DCA treated samples contained the most inflammation while PDC/DCA contained minor edema and fewer inflammatory markers compared to DCA alone. These findings are consistent with the previous study, suggesting again that PDC on its own causes minimal inflammation and may be protective while PDC/DCA and DCA alone do induce inflammatory necrosis (40). In contrast to the first two studies, recent morphologic analysis of mice inguinal fat pad tissue injected with PDC show “noticeable signs of inflammation and tissue necrosis” (41).

This comprehensive review of morphologic findings after injection of DCA and PDC individually consistently concludes that DCA and not PDC is the main bioactive component. A few studies suggest that the mode of action of DCA is necrosis as opposed to apoptosis and many, but not all (35) of the studies, assume that DCA acts non-selectively on different types of tissue. Multiple studies on PDC alone conclude that PDC may cause a minimal amount of inflammation or is protective against the effects of DCA. In the next section of this review, we will elaborate on the biochemical studies which attempt to clarify the singular roles of PDC, DCA, and PDC/DCA compounded.

## Cell Death

The expansion of adipose tissue can be driven either by adipocyte size (hypertrophy) or the number of adipocytes (hyperplasia) (42). Conversely, the destruction of fat can occur in two ways: induction of the lipolytic pathway to shrink the fat reserves in adipocytes, and destruction of adipocytes (cell death). When discussing cell death pathways, it is important to differentiate between the two main types of cell death: apoptosis and necrosis. Apoptosis is an intrinsic and organized cellular process. Importantly, apoptosis produces minimal inflammation and is associated with characteristic marker gene expressions (a family of proteases known as caspases). A subgroup of caspases known as effector caspases cleave target proteins to induce apoptosis and can be measured as biomarkers of apoptosis (43). Necrosis describes nonapoptotic cell death and pathologists use it to designate the presence of dead cells. As opposed to apoptosis, necrosis is largely considered to be uncontrolled, inflammatory, and associated with cell swelling (44). Necrosis is identified through histology, MTT assays, direct staining, and genetic markers of inflammation. Additionally, while apoptosis is predominantly characterized by elimination of the apoptotic cell by heterolytic degradation after phagocytosis, an alternative outcome is secondary necrosis (45). Necroptosis and pyroptosis are examples of secondary necrosis and are each characterized by unique markers (46). Necroptosis is characterized by RIP1, RIP3, and MLKL, while pyroptosis is characterized by the presence of IL-1β and IL-18 with flattened cytoplasm (47). While morphologic findings seem to indicate that these compounds induce some form of cell death, in this next section we will explicate the specific mechanisms that each utilizes in this process.

### DCA Causes Necrosis

In our search, we found 12 papers exploring the relationship between DCA and cell death. Of those papers, all concluded that DCA induces necrosis. Rotunda et al. wrote the first paper that identified a detergent property of DCA as the mechanism for fat reduction in DCA/PDC formulas. The group first determined cell viability with an MTS assay and found a sharp decrease in viability when cells were treated with 0.05% and 0.5% concentrations. The MTS and MTT assays are colorimetric assays that determine cell viability by measuring cell metabolic activity. Using a calcein dye release assay, they concluded that DCA acts as a detergent that causes cell lysis (48). Using the MTT assay, many sources confirmed that DCA reduces cell viability (33, 35, 39, 49–52). Other methods such as Oil Red-O, glycerol, triglyceride, and lactate dehydrogenase assays have also confirmed the detrimental impact DCA has on cell viability (34, 39). To obtain more specific information regarding cell death, five papers explored pro-inflammatory markers after DCA injection including TNF-α, and MCP-1 (36, 40, 49, 51, 53) Notably, Won et al. found significant increases in IL-1β, IL-6, and a significant decrease in DNA mass in cells treated with DCA (53). The doses used in these papers range from 0.03% (m/v%) to 0.5% (m/v%) with the lowest effective dose being 0.1% (m/v%). With this overwhelming support of literature and the consistent morphologic observation of cell swelling, as opposed to flattening, we conclude that DCA causes necrosis through lysing of the cell membrane (a detergent-like effect) with an accompanying inflammatory cascade.

### PDC Causes Cell Death

While the effect of DCA on cells is unanimously reported as necrosis caused by cell membrane lysis, the effect of PDC is more contested in the literature. In our search, we found eight papers that explore the relationship between PDC and cell death. Of the eight papers, two concluded that PDC does not induce cell death, while six concluded that it does.

In the two studies that determined that PDC does not cause cell death, two different approaches were used. Klein et al. determined no change in cell viability in 3T3-L1 adipocytes using the MTT assay (50). Additionally, Chung et al. took an *in vivo* approach and injected PDC into the fat pads of mice. They found 1) no change in fat pad size, and 2) no increase in inflammatory markers (40). It is important to note that the above-mentioned studies had treatment times of only four hours. In the subsequent studies indicating that PDC does induce cell death, a treatment time of at least 24 hours was used which may explain the contradictory results.

Of the six papers that concluded PDC does cause cell death, four papers first determined cell viability with the MTT assay (49, 51–54). Palumbo et al. used Oil Red-O and acridine orange/ethidium bromide staining and their data showed a change in the cell viability of primary cells treated with PDC compared to adipocytes that did not (39). To further determine the type of cell death induced by PDC, four papers explored apoptotic markers in the PDC-treated cells (49, 51, 52, 54). Li et al. found that PDC upregulated many apoptotic markers: Bax, caspase-8, caspase-9, and cleaved caspase-3 compared to the DCA and PDC/DCA formulations which did not activate apoptotic pathways, and instead induced cell membrane lysis and necrosis (52). Cleaved caspase-3 was further studied and the presence of the apoptotic marker has been consistently detected (49, 51). Evidence of pro-inflammatory markers, notably IL-1β, IL-6, and TNF-α levels were increased in PDC-treated cells (53, 54). Moreover, Jung et al. found that inhibition of TNF-α in PDC-treated adipocytes, significantly inhibited apoptosis as measured by caspase-3 and MTT assays, strongly suggesting that PDC induces apoptosis using a TNF-α dependent pathway (55). Their results further suggested that PDC causes lipolysis in adipocytes through a TNF-α dependent pathway (54). This will be further addressed in the subsequent section.

Combining histologic and biochemical assays, it is clear from the literature that DCA induces necrosis *via* a detergent-like effect with an accompanying inflammatory cascade. Based on morphologic findings, this effect occurs as early as one hour after treatment (34). The literature also shows that PDC induces apoptosis with minimal inflammation (seen in morphologic studies of PDC alone) but more detail explicating this process and its specific impact on adipocytes will be described in the next section.

## Lipolysis

Other than cytotoxicity (necrosis or apoptosis), another potential mechanism by which PDC or DCA may reduce adipose tissue is through adipocyte metabolism *via* the highly active lipolysis pathway. Lipolysis is defined as the metabolic pathway through which triacylglycerols stored in adipose tissue are hydrolyzed into glycerol and fatty acids. Several lipases and proteins regulate this process. Hormone-sensitive lipase (HSL) is a diffusely distributed multifunctional enzyme found in the adipocyte cytosol in unstimulated conditions. When lipolytic pathways are stimulated, HSL is translocated to the surface of perilipin-coated lipid droplets. Through the phosphorylation of perilipin, the droplets are fragmented and dispersed. Additionally, adipose triglyceride lipase (ATGL) plays a role in the dispersion of the perilipin-coated lipid droplets with a similar mechanism. After release from the perilipin-coated droplets, monoacylglycerol lipase (MGL) catalyzes the final step of triglyceride hydrolysis. During the process of lipolysis, glycerol moves across the cell membrane by facilitated diffusion and is often used as a marker of lipolysis. This pathway is regulated by various upstream regulators. The most studied pathway involves the activation of adrenergic receptors by catecholamines that stimulate adenylyl cyclase and promote cAMP production and cause subsequent lipolysis. However, other factors such as TNF-α and IL-6 can induce delayed lipolytic stimulation. While the exact mechanism of TNF-α induced lipolysis is not fully known, there is repeated evidence of lipolysis after continued stimulation with TNF-α (56). Below we will discuss the effect of DCA and PDC on lipolysis.

### DCA Does Not Cause Lipolysis

Our review identified three papers that addressed DCA’s role in lipolysis. Klein et al. studied PDC, DCA, and PDC/DCA on 3T3L-1 adipocytes using a glycerol release assay and did not find induction of lipolysis by any of these components (50). Won et al. studied gene expression of lipolysis-related genetic markers, and concluded, like Klein et al., that DCA does not induce lipolysis. Specifically, they found that DCA inhibits lipolytic markers: HSL, perilipin, ATGL, and triacylglycerol hydrolase (TGH). Supporting this, morphologic evidence of lipolysis was not seen in tissue injected with DCA compared to the positive control (53). More recently, Schmid et al. also utilized glycerol assays and found that DCA inhibits the TNF-*α* mediated pathway of lipolysis in 3T3L-1 adipocytes (57).

### PDC Induces Lipolysis Through a TNF-α Mediated Pathway

To our knowledge, the first paper that suggested that PDC may induce lipolysis was in 2003 by Young. They note that patients injected with PDC often report itching and erythema which might be due to histamine release which is associated with lipase activity (2). More mechanistic studies were published in later years. Won et al. found that, unlike DCA, PDC does induce lipolysis by increasing the gene expression of HSL (53). In 2018, Jung et al. studied the mechanism of PDC using differentiated 3T3L-1 adipocytes and mice. They found that PDC utilizes a TNF-α dependent pathway to induce lipolysis in both models. TNF-α has been shown to induce lipolysis in other studies as well (58–61). They also found that the NFκB promoter region was responsible for TNF-α release and by inhibiting both NFκB and TNF-α, lipolysis was inhibited. Results were quantified using glycerol assays as well as measuring gene expression of lipolysis-related genes. Like Won et al., Jung et al. found an increase in expression of HSL in PDC-treated mice. Interestingly, they also studied high-fed diet (HFD) mice treated with PDC and found significant suppression of lipogenic genes such as FAS, PPAR-γ, and GLUT4 (54). In 2019, Jung et al. again published results that confirmed PDC’s role in inducing TNF-α dependent lipolysis. In this study, IL-1β was similarly found to be responsible for PDC-induced lipolysis. They also found that PDC targeted adipocytes in contrast to fibroblasts, skeletal muscle cells, and endothelial cells (55). Multiple studies show that TNF-α induces apoptosis in adipocytes (62–64). Additionally, papers by Green et al., Gasic et al., Sethi et al., and Okazaki et al. have shown that TNF-α alone induces lipolysis independent of the well-known adrenergic receptor pathway (58–61). Thus, it is conceivable that after a certain amount of time, PDC induces the release of TNF-α which leads to subsequent activation of the lipolysis pathway.

Some papers oppose the above theory and suggest that PDC does not induce lipolysis. The earliest paper we found studying this was in 1977 by Prosdocimi et al. They found that PDC inhibited adrenergic receptor-mediated lipolysis but did not inhibit basal levels of lipolysis (65). Klein et al.’s study in 2009, found that similar to DCA and PDC/DCA, PDC alone does not induce lipolysis through an enzymatic pathway based on studies with 3T3L-1 adipocytes and glycerol release assays (50). These results may contradict our proposed model, however, the cells were incubated for four hours, which is significantly less than Wong et al.’s (7 days) and Jung et al.’s (24 hours). Since our hypothesis is predicated on the notion that PDC induces apoptosis, which then, through the release of TNF-α, induces lipolysis, there must be an appropriate length of exposure to the agent. Kim et al. found that eight-hour PDC treatment did not cause lipolysis in 3T3-L1 adipocytes (49). This paper is consistent with our model because at that point, cell death did occur, as our theory suggests, but there was not enough time for the TNF-α induced lipolysis pathway to occur which most likely occurs after at least 24 hours. The same is true for a more recent study by Kim and Chung which studied cells after only three hours of incubation (41).

In summary, based on the current literature, it appears that while DCA does not induce lipolysis, PDC induces lipolysis through the release of TNF-α and subsequent apoptosis after at least 24 hours of incubation.

## Combined Effects of PDC/DCA

While many studies compare the separate mechanisms of action of PDC and DCA on fat reduction, several investigators have studied the combined effect of both compounds. Our review found four articles that report a differential or synergistic effect of the two compounds.

### Differential Effects of PDC/DCA on Adipocytes and Stromal Cells

Human adipose tissue has been shown to be cellularly heterogeneous *via* single-cell resolution (66). As such, one group assessed the differential timing of cell lysis by treating cells in adipose tissue with PDC/DCA: preadipocytes, vascular smooth muscle cells, skeletal muscle myotubes, renal epithelial cells, and mature adipocytes. Interestingly, they demonstrated that mature adipocytes and differentiated lipid-laden adipocytes were more resistant to the cytolytic effects than other cell types (67).

### PDC and DCA Interaction

Other groups have compared the effects of PDC, DCA, and a combined formulation and reported an interaction between the two components. Authors have separately suggested that a unique sequence of bioactive events or different dispersion properties of the two components synergistically are responsible for the reduction of fat. Additionally, as referenced earlier, one paper suggests attenuation of DCA’s inflammation by PDC.

Palmer et al. were the first to describe a hypothesis for a synergistic sequence of bioactive events in which PDC, DCA, and benzyl alcohol initially cause cell membrane disruption. However, once injected into the subcutaneous layer, PDC is then responsible for a chain of reactions over the treatment duration. This hypothesis is yet to be proven. Similarly, other groups showed that a critical concentration of PDC is necessary to destabilize the adipocyte cell membrane which causes a complex enzymatic cascade involving the release of lipases as well as a rapid apoptotic cascade. If the critical concentration is not reached, the unstable cell membrane forms gaps or pores, allowing efflux of some of the cytoplasmic contents (68, 69).

As referenced earlier, Gupta et al. showed that the differential dispersion qualities of PDC and DCA work together to cause fat reduction. At lower concentrations, PDC/DCA causes non-specific cell death and is more effective in adipocyte death over a wider area than DCA alone. Mechanistically, the dispersive action of PDC, which spreads over a wider area synergistically works with DCA which remains locally in the injected site. At higher clinical concentrations DCA alone is as effective as PDC/DCA and remains pooled at the injection site (33).

Palumbo et al. compared PDC, DCA, and PDC/DCA on primary adipocytes and human adipose tissue. As discussed earlier, microscopic observation of adipocytes showed that PDC may play a protective role against the DCA-induced adipocyte lysis (39).

## Conclusion

**Figure 1:**
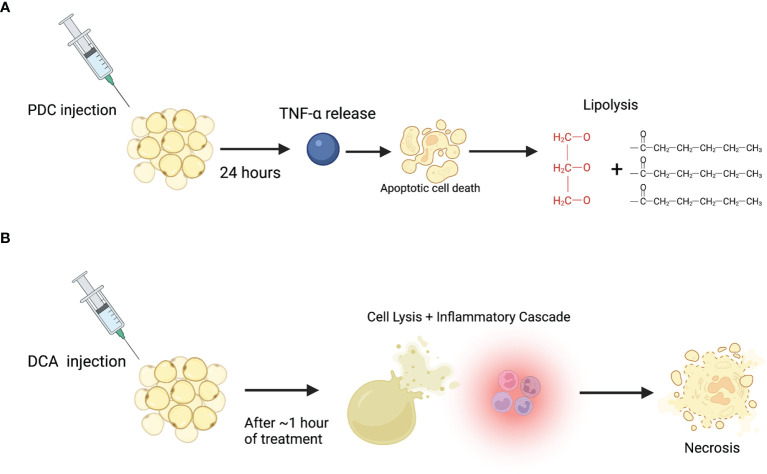
Proposed mechanism of action for PDC and DCA on adipocytes. **(A)** PDC injection induces TNF-α release which leads to apoptosis and subsequent enzymatic lipolysis after at least 24 hours post-injection. **(B)** DCA injection induces immediate cell lysis (through a detergent-like effect), accompanying inflammation, and subsequent necrosis.

In conclusion, morphologic and biochemical studies have consistently shown that DCA acts as a detergent inducing cell lysis and subsequent necrosis while PDC induces TNF-α, apoptosis, and consequent lipolysis (**Figure 1**). Several studies attempted to understand the synergistic or differential properties of PDC/DCA, but more studies are needed that compare all three compounds for longer periods. This review was limited by its focus on biochemical and cellular assays and did not review the literature that explore the clinical effects of these compounds. However, clinicians should be aware of the lack of a clear consensus surrounding the mechanisms of these drugs and consider that when potentially treating patients with these agents. Additionally, as PDC may be the source of enzymatic lipolysis and morphologic studies conclude that it is minimally inflammatory or protective against the necrotizing impacts of DCA, a more expansive role in fat reducing agents should be re-explored. Moreover, its role outside of cosmetic fat reduction should be considered, specifically for the treatment of obesity and lipomas. The high prevalence of obesity is well documented in the literature. Data from the National Health and Nutrition Examination Surveys (NHANES) found that in the United States, 35% of adults are obese and almost 30% of children or adolescents are either obese or overweight (70). A disheartening recent article published in the New England Journal of Medicine suggested that by 2030, almost 50% of adults will have obesity and nearly 25% will have severe obesity (71). Obesity is highly co-morbid with other severe diseases including specific cancers, cardiovascular disease, stroke, osteoarthritis, sleep apnea, and hypertension. Additionally, it is highly associated with all-cause mortality (70). Despite its known prevalence and severity, few effective non-surgical options for the treatment of obesity exist. Our study shows that PDC is minimally inflammatory and can reduce fat through a controlled, enzymatic, lipolytic pathway, making it a promising potential treatment for obesity. It is also important to re-explore its role in lipoma reduction, especially for patients with familial lipoma conditions like Dercum disease and familial multiple lipomatosis. These diseases predispose affected individuals to the development of up to thousands of lipomas making surgical excision impractical (72). Notably, oral PDC is already available as an over-the-counter supplement and is “generally recognized as safe” by the FDA with no carcinogenicity associated with it (73). Similarly, injectable DCA is FDA approved with no concerns for carcinogenicity, making both compounds potentially viable for therapeutic uses as described above. Further directions for research include characterizing the molecular, biochemical, and signaling basis of PDC-induced lipolysis to design a more selective and potent injectable fat reducing compound. Additionally, it is important to study the cellular trajectory of necrosed adipocytes secondary to DCA injection and explore their regenerative potential.

## Author Contributions

AM, MP, and YK drafted the manuscript. BM and KS helped draft and edit the manuscript. All authors have read and agreed to the submitted version of the manuscript.

## Funding

This study was partially supported by the NIH (DK110426). We also would like to thank the Department of Medicine for the start-up funding.

## Conflict of Interest

The authors declare that the research was conducted in the absence of any commercial or financial relationships that could be construed as a potential conflict of interest.

## Publisher’s Note

All claims expressed in this article are solely those of the authors and do not necessarily represent those of their affiliated organizations, or those of the publisher, the editors and the reviewers. Any product that may be evaluated in this article, or claim that may be made by its manufacturer, is not guaranteed or endorsed by the publisher.

## Abbreviations

DCA, Deoxycholic Acid; PDC, Phosphatidylcholine; TNF-α, Tumor Necrosis Factor-alpha; SMF, Submental fat.
